# Periventricular and juxtacortical characterization of UManitoba-JHU functionally defined human white matter atlas networks

**DOI:** 10.3389/fnhum.2023.1196624

**Published:** 2023-07-06

**Authors:** Chase R. Figley, Teresa D. Figley, Kaihim Wong, Md Nasir Uddin, Rodrigo Dalvit Carvalho da Silva, Jennifer Kornelsen

**Affiliations:** ^1^Department of Radiology, University of Manitoba, Winnipeg, MB, Canada; ^2^Division of Diagnostic Imaging, Health Sciences Centre, Winnipeg, MB, Canada; ^3^Neuroscience Research Program, Kleysen Institute for Advanced Medicine, Winnipeg, MB, Canada; ^4^Department of Physiology and Pathophysiology, University of Manitoba, Winnipeg, MB, Canada; ^5^Department of Neurology, University of Rochester, Rochester, NY, United States; ^6^Department of Biomedical Engineering, University of Rochester, Rochester, NY, United States

**Keywords:** human, brain, cortex, ventricle, white matter, structural connectivity, periventricular, juxtacortical

## Abstract

**Background:**

The open-access UManitoba-JHU functionally defined human white matter (WM) atlas contains specific WM pathways and general WM regions underlying 12 functional brain networks in ICBM152 template space. However, it is not known whether any of these WM networks are disproportionately co-localized with periventricular and/or juxtacortical WM (PVWM and JCWM), which could potentially impact their ability to infer network-specific effects in future studies—particularly in patient populations expected to have disproportionate PVWM and/or JCWM damage.

**Methods:**

The current study therefore identified intersecting regions of PVWM and JCWM (defined as WM within 5 mm of the ventricular and cortical boundaries) and: (1) the ICBM152 global WM mask, and (2) all 12 UManitoba-JHU WM networks. Dice Similarity Coefficient (DSC), Jaccard Similarity Coefficient (JSC), and proportion of volume (POV) values between PVWM (and JCWM) and each functionally defined WM network were then compared to corresponding values between PVWM (and JCWM) and global WM.

**Results:**

Between the 12 WM networks and PVWM, 8 had lower DSC, JSC, and POV; 1 had lower DSC and JSC, but higher POV; and 3 had higher DSC, JSC, and POV compared to global WM. For JCWM, all 12 WM networks had lower DSC, JSC, and POV compared to global WM.

**Conclusion:**

The majority of UManitoba-JHU functionally defined WM networks exhibited lower than average spatial similarity with PVWM, and all exhibited lower than average spatial similarity with JCWM. This suggests that they can be used to explore network-specific WM changes, even in patient populations with known predispositions toward PVWM and/or JCWM damage.

## Introduction

The UManitoba-JHU functionally defined human white matter (WM) atlases (Figley et al., 2015, 2017) are an open-access resource^1^ for investigating specific WM pathways and/or general WM regions underlying 12 previously reported (Shirer et al., 2012) intrinsic functional brain networks.^2^ These include: dorsal Default Mode Network (dDMN), ventral Default Mode Network (vDMN), left Executive Control Network (lECN), right Executive Control Network (rECN), anterior Salience Network (aSN), and posterior Salience Network (pSN), Precuneus Network (PN), Language Network (LN), Basal Ganglia Network (BGN), Higher Visual Network (HVN), Visuospatial Network (VSN), and Sensorimotor Network (SMN).^3^ Analogous to gray matter (GM) region-of-interest (ROI) functional connectivity analyses of task- or resting-state functional MRI (fMRI) (van den Heuvel and Hulshoff Pol, 2010; Cole et al., 2014), these WM atlases can be used with various types of quantitative MRI methods (e.g., diffusion MRI, magnetization transfer imaging, T1w/T2w ratio, myelin water imaging, etc.) (Geeraert et al., 2018; Uddin et al., 2019) to examine network-based structural connectivity differences between individuals or groups (e.g., related to behavioral measures in neurologically healthy populations or clinical characteristics/outcomes in various patient populations).

However, although the original papers describing the UManitoba-JHU WM atlases (Figley et al., 2015, 2017) reported parameters such as the total WM volume ascribed to each network and the amount of overlap between different WM networks, they did not examine whether (or to what extent) any of these networks might have different spatial similarities or contain different proportions of periventricular white matter (PVWM) and/or juxtacortical white matter (JCWM) compared to global WM—which could be an important consideration when studying certain patient populations with known proclivities to PVWM and/or JCWM damage, such as multiple sclerosis (MS) (Filippi et al., 2019; Bouman et al., 2021; Gauthier, 2022; Pirpamer et al., 2022; Tonietto et al., 2022; Vaneckova et al., 2022), cerebral small vessel disease (Phuah et al., 2022; van Veluw et al., 2022), microbleeds in mild traumatic brain injury (Hageman et al., 2022), and NOTCH3-related spontaneous intracerebral hemorrhages (Chen et al., 2022).^4^

For example, let us consider a hypothetical experiment seeking to determine whether MS patients (on average) have disproportionate amounts of WM loss and/or microstructural damage within the dDMN compared to other WM regions. In principle, these types of questions can be addressed using the UManitoba-JHU WM networks by either spatially normalizing each patient’s brain MRI data to the template (and working in template space) or by applying a patient-specific inverse spatial normalization to the template (and working in subject space) (Pirzada et al., 2020). Either way, let us say that: (1) the UManitoba-JHU dDMN WM mask was used to compare a sample of participants with MS to a matched sample of healthy controls; and (2) that the dDMN WM regions were found to have disproportionately lower volumetric and microstructural values (i.e., statistically significant results after correcting for any unmatched variables, family wise errors owing to multiple comparisons, etc.). In this case, it might be tempting to conclude that there is a network-specific effect, in which MS appears to disproportionately target dDMN WM. However, since it is well established that MS disproportionately affects PVWM and JCWM regions, what if the dDMN WM mask itself was made up of exclusively (or at least disproportionately) PVWM and JCWM regions? Although it would not invalidate the statistically significant findings *per se*, such a confound would make it difficult (if not impossible) to conclude definitively whether the difference was driven by PVWM and/or JCWM damage, or whether perhaps there was something more inherently interesting about the dDMN network itself.

To address this issue, the aim of the current manuscript is to characterize the spatial similarity between, as well as the proportion of PVWM and JCWM within each of the UManitoba-JHU functionally defined human WM networks, and to compare these to the corresponding PVWM and JCWM measures in global WM to determine whether (and if so, to what extent) any such confounds may exist.

## Methods

Since the UManitoba-JHU WM networks are all spatially normalized to the ICBM152 template (Mazziotta et al., 2001a,b), global PVWM and JCWM masks (and the resulting volumes of each) will be derived from the T1-weighted single-subject ICBM152 image included with the UManitoba-JHU atlas (Figley et al., 2015, 2017)―which is an interpolated (1.00 mm^3^) version of the single-subject ICBM152 image distributed with SPM8.^5^ Once the PVWM and JCWM masks have been created, they can then be compared to both the global WM mask and each of the UManitoba-JHU WM network masks to evaluate: (1) spatial similarity using the Dice Similarity Coefficient (DSC), (2) spatial similarity using the Jaccard Similarity Coefficient (JSC), and (3) the proportion of overlapping volume (POV) with respect to the global and network WM masks (Yeghiazaryan and Voiculescu, 2018; Costa, 2022). Therefore, because this study relies exclusively on publicly available brain atlases and software tools (i.e., not involving any human or animal study participants), Research Ethics Board approval is not required.

### Defining global white matter, ventricular, and cortical ROIs

**FIGURE 1:**
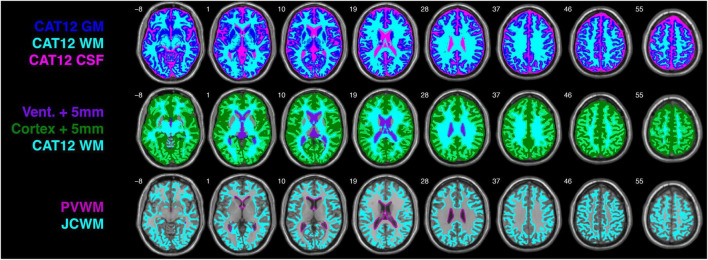
CAT12 gray matter (GM), white matter (WM), and cerebrospinal fluid (CSF) tissue segmentations from the single-subject T1-weighted ICBM152 image (top row), ventricle and cortical masks following 5 mm 3D dilation with the CAT12 WM mask (second row), and periventricular WM (PVWM) and juxtacortical WM (JCWM) masks. For the purposes of this investigation, PVWM is defined as WM within 5 mm of the ventricles, and JCWM is defined as WM within 5 mm of the cortex.

The 1.00 mm × 1.00 mm × 1.00 mm single-subject T1-weighted ICBM152 image included with the UManitoba-JHU atlas was segmented using the Computational Anatomy Toolbox (CAT12 version r1318)^6^ within the Statistical Parametric Mapping software (SPM12 version 7219).^7^ With the exception of running CAT12 in “expert mode” to enable the cerebrospinal fluid (CSF) mask to be written out along with the GM and WM masks (top row Figure 1), tissue segmentation was performed using default CAT12 parameters.

To create ventricular and cortical masks, superfluous voxels beyond these regions were removed from the whole-brain CAT12 CSF and GM masks. This was achieved using the CerebrA atlas and ROI lookup table (Manera et al., 2020), which was initially 3D dilated by 15 mm (in each direction) and then used to: (1) exclude CSF voxels outside of the bilateral third ventricles, lateral ventricle, and inferior lateral ventricle ROIs (for the ventricle mask); and (2) exclude GM voxels from cerebellar and subcortical ROIs (for the cortical mask).

### Defining periventricular and juxtacortical white matter ROIs

Although specific definitions of “periventricular” and “juxtacortical” WM differ (Barkhof and Scheltens, 2006), recent measurements from concentric periventricular bands have shown that quantitative magnetization transfer ratios rapidly increase between 1 and 4 mm from the ventricle boundary, and then become stable after a distance of 5 mm among neurologically healthy controls, as well as patients with clinically isolated syndrome and relapsing-remitting MS (Pirpamer et al., 2022). For the purposes of the current study, we therefore chose an *a priori* definition for PVWM as WM within 5 mm of the ventricle mask, and JCWM as WM within 5 mm of the cortical mask. Thus, the ICBM152 ventricle and cortical masks were 3D dilated by 5 mm and overlaid on the global WM mask (middle row Figure 1), such that overlapping regions yielded PVWM and JCWM masks (bottom row Figure 1).

### Evaluating spatial similarity

In order to determine whether any of the WM networks shared higher spatial similarity with PVWM or JCWM (compared to the spatial similarities between global WM and PVWM or JCWM), we calculated three common similarity metrics; namely, the Dice Similarity Coefficient (DSC; a.k.a., Sørensen–Dice Index or F1 Score), the Jaccard Similarity Coefficient (JSC; a.k.a., Jaccard Index or Intersection over Union), and the Proportion of Overlapping Volume (POV; a.k.a., True Positive Volume Fraction) (Yeghiazaryan and Voiculescu, 2018; Costa, 2022). Where A is the set of elements in the PVWM (or JCWM) mask and B is the set of elements in each WM ROI (i.e., either the global WM mask or each of the 12 functionally defined WM network masks), and where ∩ is the mathematical operator for intersection (i.e., overlapping elements between sets) and ∪ is the mathematical operator for union (i.e., all elements within the collection of sets): (1) the DSC is defined as twice the number of intersecting (overlapping) elements divided by the total number of elements in each of the sets (i.e., DSC${}_{A,B}=\frac{2\,\left|\text{A}\cap \text{B}\right|}{\left|\text{A}\right|+\left|\text{B}\right|}$); (2) the JSC is defined as the number of intersecting (overlapping) elements divided by the number of elements in the collection of sets (i.e., JSC${}_{A,B}=\frac{\left|\text{A}\cap \text{B}\right|}{\left|\text{A}\cup \text{B}\right|}=\frac{\left|\text{A}\cap \text{B}\right|}{\left|\text{A}\right|+\left|\text{B}\right|-\left|\text{A}\cap \text{B}\right|}$); and (3) the POV is defined as the number of intersecting (overlapping) elements divided by number of elements in the ROI of interest (i.e., POV${}_{A,B}=\frac{\left|\text{A}\cap \text{B}\right|}{\left|\text{B}\right|}$) (Yeghiazaryan and Voiculescu, 2018; Costa, 2022). Therefore, using the PVWM and dDMN as an example: DSC${}_{\mathrm{PVWM},\mathrm{dDMN}}=\frac{2\,\left|\text{PVWM}\cap \text{dDMN}\right|}{\left|\text{PVWM}\right|+\left|\text{dDMN}\right|}$, JSC${}_{\mathrm{PVWM},\mathrm{dDMN}}=\frac{\left|\text{PVWM}\cap \text{dDMN}\right|}{\left|\text{PVWM}\right|+\left|\text{dDMN}\right|-\left|\text{PVWM}\cap \text{dDMN}\right|}$, and POV${}_{\mathrm{PVWM},\mathrm{dDMN}}=\frac{\left|\text{PVWM}\cap \text{dDMN}\right|}{\left|\text{dDMN}\right|}$.

**FIGURE 2:**
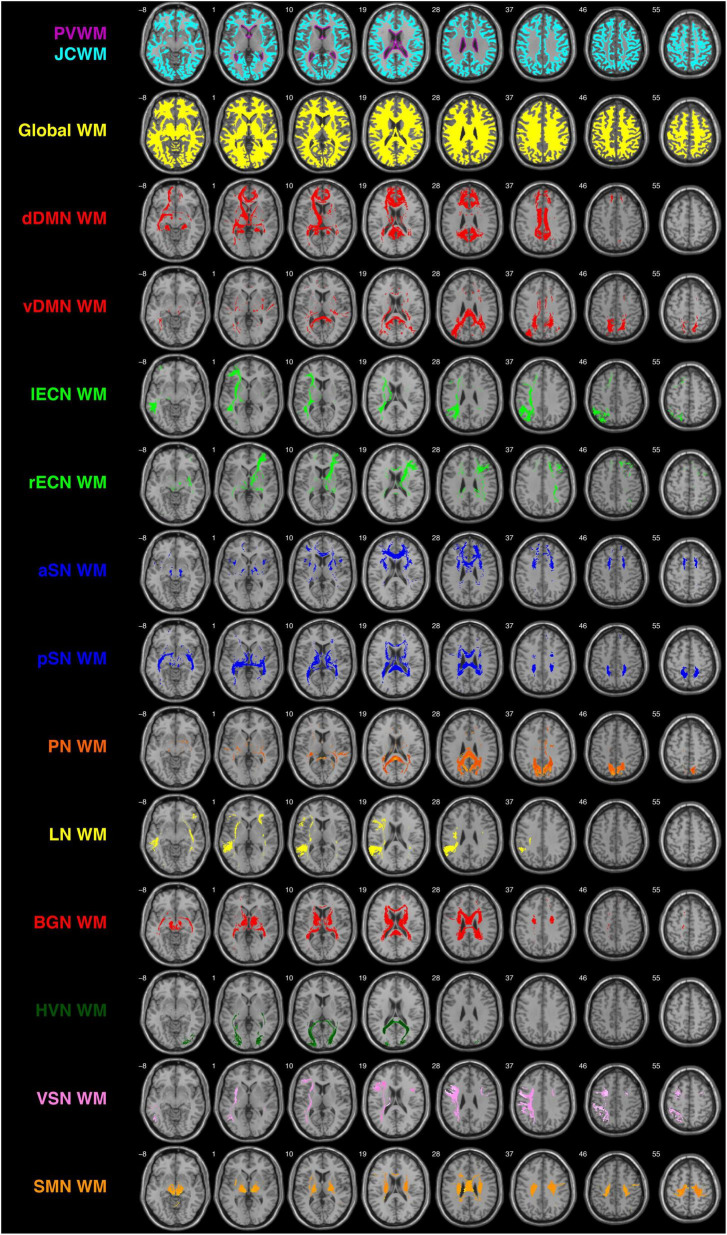
Depictions of the PVWM and JCWM masks (top row), global WM mask (second row), and each of the 12 functionally defined human white matter networks investigated in the current study (rows 3–14) overlaid on the single-subject T1-weighted ICBM152 template. dDMN, dorsal default mode network; vDMN, ventral default mode network; lECN, left executive control network; rECN, right executive control network; aSN, anterior salience network; pSN, posterior salience network; PN, precuneus network; LN, language network; BGN, basal ganglia network; HVN, higher visual network; VSN, visuospatial network; SMN, sensorimotor network.

Having already created the ICBM152 PVWM and JCWM masks (Figure 2 Row #1), the global WM mask (Figure 2 Row #2), and each of the network WM masks (Figure 2 Row #3-14), the DSC, JSC, and POV values were computed between PVWM (and JCWM) and each of the other WM ROIs, allowing the network WM metrics to be directly compared to the corresponding global WM values. Although DSC, JSC, and POV measure similarity in slightly different ways, they all scale between 0 and 1, which (within the context of 3D binary ROIs like the PVWM, JCWM, global WM, and each of the UM-JHU network WM masks) corresponds to having no spatial similarity and perfect spatial similarity, respectively.

## Results and discussion

Based on a proximity within 5 mm of the ventricles of the ICBM152 template, PVWM was found to account for 15,429 mm^3^ (1.79%) of the 862,542 mm^3^ global WM ROI. Table 1 lists the total volume, PVWM volume, as well as the DSC, JSC, and POV spatial similarity measures between PVWM and each of the other WM ROIs (i.e., global WM and each of the WM networks in the UManitoba-JHU atlas). Out of the 12 WM network masks, 8 had uniformly lower spatial similarity scores (i.e., lower DSC, JSC, and POV) with PVWM compared to global WM. These included the dDMN, vDMN, lECN, aSN, PN, LN, VSN, and SMN. Among the remaining WM networks, 3 (i.e., the pSN, BGN, and HVN) were found to have uniformly higher spatial similarity scores; and one WM network (i.e., the rECN) was found to have lower DSC and JSC, but a slightly higher POV.

**TABLE 1:** Total ROI volume, volume of periventricular white matter (PVWM) within ROI, Dice similarity coefficient (DSC), Jaccard similarity coefficient (JSC), and proportion of overlapping volume (POV) of PVWM within global white matter and each network WM mask.

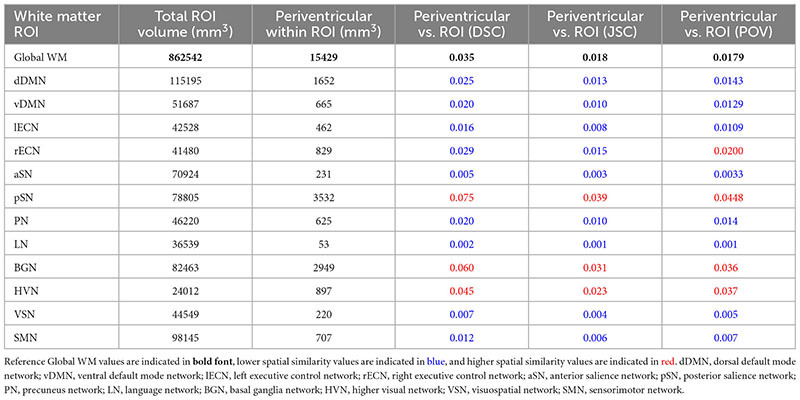

Based on a proximity within 5 mm of the cortex of the ICBM152 template, JCWM was found to account for 480,290 mm^3^ (55.68%) of the 862,542 mm^3^ global WM ROI. Table 2 lists the total volume, JCWM volume, as well as the DSC, JSC, and POV spatial similarity measures between JCWM and each of the other WM ROIs (i.e., global WM and each of the WM networks in the UManitoba-JHU atlas). In this case, all 12 of the WM networks were found to have uniformly lower spatial similarity scores (i.e., lower DSC, JSC, and POV) with JCWM compared to global WM.

**TABLE 2:** Total ROI volume, volume of juxtacortical white matter (JCWM) within ROI, Dice similarity coefficient (DSC), Jaccard similarity coefficient (JSC), and proportion of overlapping volume (POV) of JCWM within global white matter and each network WM mask.

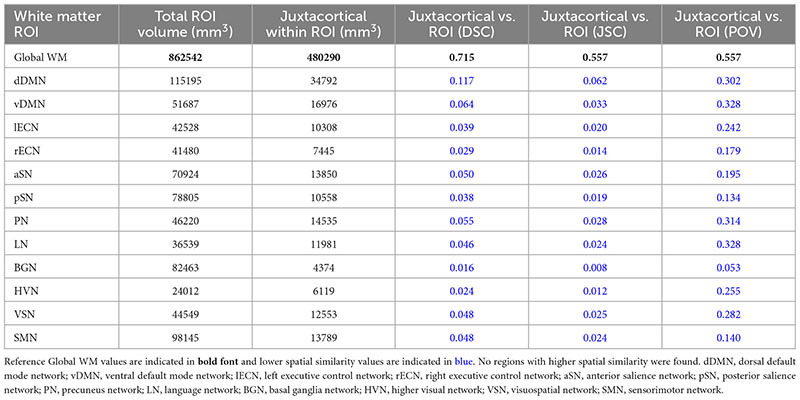

One potentially interesting tangential observation is that, within the same 5 mm distance from the cortical and ventricular boundaries, there appears to be approximately 31 times the amount of JCWM (55.68%) compared to PVWM (1.79%) in the overall WM mask. Such a large disparity may initially seem surprising, but is likely the result of two factors: (1) the much larger surface area of the cortical sheet, compared to the ventricular boundary;^8^ and (2) the relatively large amount of competing periventricular GM (e.g., bilateral caudate and thalamus), which means that the already smaller tissue volume within 5 mm of the ventricles is distributed between WM and GM.

Nonetheless, to account for the disparity between PVWM and JCWM volumes, *post hoc* analyses to assess the spatial similarity across the combined PVWM and JCWM masks were computed for global WM and the four networks that showed higher than average spatial similarity relative to PVWM in Table 1 (i.e., rECN, pSN, BGN, and HVN). Perhaps not surprising given the results reported in Table 2 and the disparity between total PVWM and JCWM volumes, the combined PVWM and JCWM analysis revealed that all three spatial similarity metrics were higher for global WM (DSC = 0.730, JSC = 0.575, and POV = 0.575) compared to any of the network ROIs (highest DSC = 0.049 for pSN, highest JSC = 0.025 for pSN, and highest POV = 0.292 for HVN).

We therefore make the following recommendations for future studies aiming to use the UManitoba-JHU WM networks to infer network-based differences and/or changes:

1. For study populations with no predispositions to PVWM or JCWM damage: All 12 networks can likely be used to draw network-based inferences.
2. For study populations with known predispositions to PVWM damage: Network-based inferences can likely be drawn regarding the dDMN, vDMN, lECN, aSN, PN, LN, VSN, SMN without any apparent spatial confounds with PVWM regions. However, any network-based inferences regarding the pSN, BGN, and HVN should be discussed within the context of the inherent spatial confounds. Similarly, although the rECN network had lower DSC and JSC, network-based inferences should likely be discussed within the context of the marginally higher POV with PVWM.
3. For study populations with known predispositions to JCWM damage: All 12 networks can likely be used to draw network-based inferences without any apparent spatial confounds with JCWM regions.
4. For study populations with known predispositions to both PVWM and JCWM damage: All 12 networks can likely be used to draw network-based inferences without any spatial confounds with the combined PVWM and JCWM mask. However, to be forthright, any network-based inferences regarding the pSN, BGN, and HVN should likely be qualified and discussed within the context of their spatial confounds with PVWM.

It should be noted that many factors can influence overall and regional brain volumes, as well as the proportional volumes of different tissue compartments. However, while there are well known sex differences in total brain, GM, WM, and CSF volumes, previous studies have shown that males and females have similar proportions of WM (relative to total brain volume) (Lüders et al., 2002), and that normal appearing cerebral WM volumes tend to scale quite linearly with (and are arguably one of the best predictors of) total brain volume within and between sexes (Jäncke et al., 2019). This suggests that the current findings, with respect to the PVWM and JCWM proportions of the UManitoba-JHU WM atlases, should be broadly generalizable. However, a more recent study suggesting that WM volume scaling is regionally heterogeneous (with larger brains having disproportionately higher WM volumes in the genu and splenium of the corpus callosum, as well as anterior and posterior cortical regions) (Warling et al., 2021) suggests that there could be exceptions for certain patient groups, and presumably in very young (pediatric) or very old (geriatric) populations.

Finally, although the current findings are specific to the UManitoba-JHU WM atlas ROIs reported above, the same approach and methodology could be employed in future studies to examine PVWM and/or JCWM components of other WM ROIs—including those from the JHU (Mori et al., 2008; Oishi et al., 2009), HCP (Yeh et al., 2018), or other WM atlases that (to the best of our knowledge) have not been previously characterized in this way. Moreover, while most of the combined, network-level UManitoba-JHU WM ROIs were not found to disproportionately overlap with PVWM or JCWM regions, individual WM connections within these and other networks still might. Therefore, researchers employing advanced anatomical connectivity analyses—i.e., quantitative MRI values of individual white matter connections (e.g., diffusivity, streamline count, myelin water fraction), graph theoretical metrics, etc.—to infer “network difference” or “network changes” should be conscious of potential biases in studies involving patient populations with known predispositions to PVWM and/or JCWM damage.

## Conclusion

In summary, the current study characterized the spatial similarities between PVWM (and JCWM) and each of the networks in the UManitoba-JHU functionally defined human WM atlas, and compared these to PVWM (and JCWM) in global WM. The findings indicate that only 3 out of the 12 WM networks (i.e., the pSN, BGN, and HVN) had uniformly higher spatial similarity with PVWM compared to global WM, and that the majority of WM networks had uniformly lower spatial similarity. Moreover, all 12 networks were found to have uniformly lower spatial similarity with JCWM compared to global WM. Therefore, our overall conclusions are: (1) that, with the exception of PVWM in the pSN, BGN, and HVN networks, PVWM and JCWM are actually spatially under-represented in the UManitoba-JHU functionally defined WM networks; and therefore (2) that the majority of these WM networks can be used to investigate network-specific WM effects in future studies, even in patient populations with known predispositions toward PVWM and/or JCWM damage.

## Data availability statement

Publicly available datasets were analyzed in this study. This data can be found here: https://www.nitrc.org/projects/uofm_jhu_atlas/.

## Author contributions

CF and TF performed the data analysis and prepared the figures and tables. All authors contributed to the study conception, experimental design, interpretation of the data, manuscript preparation, and manuscript review (including approving the final draft of the manuscript).

## Footnotes

1
http://www.nitrc.org/projects/uofm_jhu_atlas

2
http://findlab.stanford.edu/functional_ROIs.html

3 All 14 functional networks were investigated using the same methodology, but no white matter tracts met the *a priori* thresholds for inclusion in either the Auditory Network (AN) or Primary Visual Network (PVN) (Figley et al., 2017).

4 These are just a few examples of diseases that appear to disproportionately affect PVWM and/or JCWM regions, but this list is not exhaustive.

5
https://www.fil.ion.ucl.ac.uk/spm/software/spm8/

6
http://www.neuro.uni-jena.de/cat/

7
http://www.fil.ion.ucl.ac.uk/spm/software/spm12/

8 Due to the complex folding patterns of the sulci/gyri, the surface area of an adult human neocortex is approximately 1600 cm^2^ (Van Essen and Drury, 1997).
